# Exploring the link between asprosin and glucose and lipid dysregulation in offspring of diabetic mothers in a rat model

**DOI:** 10.1371/journal.pone.0354870

**Published:** 2026-09-18

**Authors:** Hamidreza Ahmadasadi, Peyman Rasouli Emamgholi, Issa Layali, Tina Kianfar, Shahriyar Tork, Mobina Jafarpour, Masoume Aliabadi, Hafez Heydari

**Affiliations:** 1 Department of Biochemistry and Biophysics, TeMS.C., Islamic Azad University, Tehran, Iran; 2 Department of Physiology, School of Medicine, Iran University of Medical Sciences, Tehran, Iran; 3 Department of Anatomy, School of Medicine, Iran University of Medical Sciences, Tehran, Iran; 4 Department of Biochemistry, School of Medicine, Iran University of Medical Sciences, Tehran, Iran; Universidad de Murcia, SPAIN

## Abstract

Maternal diabetes mellitus (DM) is a known risk factor for offspring metabolic dysfunction, predisposing them to DM later in life. While this association is established, the molecular mechanisms underlying the intergenerational transmission of DM risk are not fully understood. This study investigated the effects of in utero exposure to maternal DM on circulating levels of asprosin in offspring, aiming to clarify the potential role of asprosin in this pathological cascade. Twelve pregnant Wistar rats were randomized into two groups: a streptozotocin (STZ)-induced diabetic group (*n* = 6) and a non-diabetic control group (*n* = 6). DM was confirmed via blood glucose measurements (>250 mg/dL) 72 hours post-STZ administration. At 16 weeks postnatal, six male offspring per group were euthanized for plasma collection. Asprosin levels were measured using a commercial ELISA kit. Statistical analyses were performed to compare group means and determine the relationship between asprosin and variables. Maternal asprosin levels were significantly higher in the DM group compared to controls (330.70 ± 78.13 vs. 244.00 ± 21.54 pg/mL, *P* = 0.02). However, offspring of both groups showed comparable circulating asprosin concentrations (234.50 ± 10.60 vs 235.00 ± 7.23 pg/mL, *P* = 0.92). In contrast to diabetic mothers, in whom asprosin was significantly correlated with atherogenic lipid parameters and glucose homeostasis indices, no significant correlations were observed between asprosin and these parameters in offspring of either group. These preliminary findings suggest that asprosin may not be a critical mediator in the intergenerational transmission of diabetes-associated metabolic dysfunction.

## 1. Introduction

Diabetes Mellitus (DM) comprises a heterogeneous group of metabolic disorders characterized by chronic hyperglycemia [1]. Individuals with DM face an increased risk of severe complications, including cardiovascular diseases (CVDs), stroke [2], neuropathy [3], retinopathy [4] and nephropathy [5]. The global prevalence of DM has increased dramatically in recent decades [6]. It is predicted that approximately 642 million adults (1 in 10) will be affected by 2045 [7].

*In utero* exposure to maternal DM is a well-established risk factor for offspring susceptibility to DM in later life [8–11]. Although the underlying mechanisms remain incompletely elucidated, emerging evidence implicates adipokines as potential mediators of intergenerational DM risk [12–14]. Offspring exposed to maternal DM exhibit persistent dysregulation of adipokine profiles [14], observable as early as infancy [15–18], which may persist into adulthood and predispose them to DM independently of obesity [19]. Notably, dysregulation of adipokines in this population correlates strongly with increased DM risk [13], underscoring their potential role in the developmental programming of diabetes. These findings support the hypothesis that aberrant adipocyte signaling and dysregulated adipokine secretion play a critical role in the intergenerational transmission of DM risk [20,21].

Asprosin, a recently identified adipokine, has been implicated in the pathogenesis of DM [22,23]. It is released from adipocytes in response to fasting and promotes rapid release of glucose from the hepatocytes [22]. Elevated circulating asprosin levels correlate with impaired insulin sensitivity [24], promote insulin resistance [25], and β-cell dysfunction [26], all hallmarks of increased DM risk in the future. Furthermore, asprosin has been linked to pro-atherogenic lipid profiles [25], suggesting a dual role in metabolic and cardiovascular pathogenesis in patients with DM. Additionally, epigenetic modifications in the *FBN1* gene, which encodes asprosin, can be induced by hyperglycemic fluctuations [27], raising the possibility that maternal DM may epigenetically reprogram offspring *FBN1* expression, thereby perpetuating DM risk across generations.

To evaluate the impact of maternal DM on offspring asprosin levels, we compared circulating asprosin levels in offspring of diabetic rats to those of offspring from normoglycemic rats. This experimental design aimed to elucidate potential associations between *in utero* hyperglycemia, offspring asprosin dysregulation, and subsequent susceptibility to DM in offspring.

## 2. Materials and methods

### 2.1. Chemicals and reagents

All analytical-grade chemicals and reagents were purchased from Sigma Aldrich (St. Louis, MO, USA), unless otherwise specified.

### 2.2. Ethics

The protocols for animal use and experimental procedures described in this study were approved by the Biomedical Research Ethics Committee of **Iran University of Medical Sciences** (Ethical code: IR.IUMS.AEC.1402.120). This approval certifies compliance with the standards established by the *Guide for the Care and Use of Laboratory Animals*.

### 2.3. Animal

Sixteen Wistar rats (including 12 females and 4 males) were used in this study. The rats, weighing 170–200 g, were obtained from the Animal House at the School of Medicine, **Iran University of Medical Sciences** (Tehran, Iran). Adult rats were acclimatized to their new environment for two weeks prior to experimentation to screen for and rule out intercurrent infections. Females were housed with normal males (three females per male) and successful mating was confirmed the following morning by vaginal smear analysis for the presence of sperm. After mating, twelve pregnant female rats were randomly assigned to two experimental groups (6 rats per group). Subsequently, pregnant females were individually housed and monitored for weight gain throughout the gestation period.

Diabetes was induced in one group (n = 6) by intraperitoneal injecting of streptozotocin (STZ; 70 mg/kg body weight) dissolved in 0.05 M citrate buffer (pH 4.5). Animals with a blood glucose level exceeding 250 mg/dl were classified as diabetic. The control group (n = 6) received citrate buffer injections.

All animals were housed in cages under controlled environmental conditions: temperature 23 ± 2 °C, adequate ventilation, 50 ± 5% relative humidity, and a 12-hours light/dark cycle (lights on at 06:00 h). They were provided with a standard rodent pellet diet and water *ad libitum*. Both diabetic and control mothers nursed their own offspring. After weaning, they were maintained on a standard rodent pellet diet and water *ad libitum* until reaching the target weight range of 170–200 g. Subsequently, each offspring was individually numbered. Finally, using a random number generation method, six male offspring were selected from the diabetic group and six male offspring from the control group. Importantly, at most one male offspring was selected from each mother for the study.

### 2.4. Sample preparation

At the end of experiment, the rats that had been subjected to 12 hours of fasting were anesthetized with 3% sodium pentobarbital and then euthanized. For each, the chest was opened, and a needle was inserted into the heart through the diaphragm to collect blood samples. Blood was drawn into tubes containing ethylenediaminetetraacetic acid (EDTA). Plasma was separated by centrifugation and stored at −80°C until further analysis.

### 2.5. Determination of biochemical profile

Glucose levels were measured using the glucose oxidase method (Pars Azmoon, Iran). Cholesterol levels were determined using the cholesterol oxidase method (Pars Azmoon, Iran). Triglyceride levels were measured using an enzymatic assay (Pars Azmoon, Iran). High-density lipoprotein cholesterol (HDL-C) levels were assessed using the precipitation method (Pars Azmoon, Iran). Low-density lipoprotein cholesterol (LDL-C) levels were calculated using the Friedewald formula. Insulin levels were measured using an ELISA kit.

The atherogenic index of plasma (AIP) was calculated as the base-10 logarithm of the ratio of triglycerides (TG) to high-density lipoprotein cholesterol (HDL-C) using the formula:


$$\begin{array}{c} AIP={log}_{10}\left(TG[mg/dL]/HDL-C[mg/dL]\right) \end{array}$$

The triglyceride-glucose (TyG) index was computed using the formula:


$$\begin{array}{c} TyG\text{ index}=ln\left[\left(TG[mg/dL]\times glucose[mg/dL]\right)/2\right] \end{array}$$

Lipid ratios and the glucose-to-insulin (G/I) ratio were derived using Microsoft Excel (version 2013, Microsoft Corp., USA), employing standard spreadsheet functions for mathematical computations.

### 2.6. Measurement of asprosin

Asprosin levels were determined using a commercial ELISA kit (ZellBio, Germany, Cat Number: ZB-11550C-H9648) according to the manufacturer’s instructions.

### 2.7. Statistical analysis

All statistical analyses were performed using STATA software. Data are reported as mean± standard deviation (SD). Differences between groups were analyzed using the independent samples *t-*test. Correlations between asprosin and biochemical parameters were assessed using the Pearson’s correlation coefficient. A *P*-value < 0.05 was considered statistically significant.

## 3. Results

### 3.1. Descriptive statistics

A comparative analysis of metabolic and lipid profiles between diabetic mothers and the control group revealed significant differences (Table 1). Blood glucose levels were markedly elevated in diabetic mothers compared to controls (253.50 ± 3.61 vs. 94.5 ± 2.16 mg/dL; *p* < 0.01). Additionally, concentrations of cardiovascular risk-associated lipids including TG, TC, LDL-C, and non-HDL-C were significantly higher in the diabetic group (154.50 ± 2.42 vs. 47.83 ± 1.72; 108.20 ± 2.63 vs. 76.00 ± 3.74; 40.43 ± 3.44 vs. 30.10 ± 3.33; and 71.33 ± 3.74 vs. 39.67 ± 3.50 mg/dL, respectively; *p* < 0.01 for all). HDL-C levels did not differ significantly between the groups (36.83 ± 3.48 vs. 36.33 ± 1.50 mg/dL; *p* = 0.75). Lipid ratios such as TC/HDL-C, TG/HDL-C, TG/LDL-C, LDL-C/HDL-C, Non-HDL-C/HDL-C and the AIP were substantially higher in diabetic mothers (2.95 ± 0.29 vs. 2.09 ± 0.11; 4.23 ± 0.49 vs. 1.31 ± 0.08; 3.84 ± 0.30 vs. 1.60 ± 0.16; 2.56 ± 0.0.56 vs. 0.98 ± 0.10; 1.95 ± 0.29 vs. 1.09 ± 0.11; and 0.62 ± 0.04 vs. 0.12 ± 0.03, respectively; *p* < 0.01 for all). Notably, insulin levels were significantly reduced in the diabetic group (0.56 ± 0.03 vs. 2.10 ± 0.07 μIU/mL; *p* < 0.01). The G/I ratio was significantly higher in diabetic mothers than in control mothers (450.5 ± 33.21 vs. 44.97 ± 1.77, *p* < 0.01). Similarly, the TyG index was also significantly elevated in the diabetic group compared to the control group (9.88 ± 0.02 vs. 7.72 ± 0.03, *p* < 0.01).

**Table 1:** Comparison of biochemical profile in diabetic and control mothers.

Parameter	Control Mothers (n = 6)	Diabetic Mothers (n = 6)	*P* value
Glucose (mg/dL)	94.50 (2.16)	253.50 (3.61)	< 0.01
TG (mg/dL)	47.83 (1.72)	154.50 (2.42)	< 0.01
TC (mg/dL)	76.00 (3.74)	108.20 (2.63)	< 0.01
HDL-C (mg/dL)	36.33 (1.50)	36.83 (3.48)	0.75
LDL-C (mg/dL)	30.10 (3.33)	40.43 (3.44)	< 0.01
Non-HDL-C (mg/dL)	39.67 (3.50)	71.33 (3.74)	< 0.01
TC/HDL-C	2.09 (0.11)	2.95 (0.29)	< 0.01
TG/HDL-C	1.31 (0.08)	4.23 (0.49)	< 0.01
TG/LDL-C	1.60 (0.16)	3.84 (0.30)	< 0.01
LDL-C/HDL-C	0.98 (0.10)	2.56 (0.56)	< 0.01
Non-HDL-C/HDL-C	1.09 (0.11)	1.95 (0.29)	< 0.01
AIP	0.12 (0.03)	0.62 (0.04)	< 0.01
Insulin (μIU/mL)	2.10 (0.07)	0.56 (0.03)	< 0.01
G/I ratio	44.97 (1.77)	450.50 (33.21)	< 0.01
TyG index	7.72 (0.03)	9.88 (0.02)	< 0.01
Asprosin (pg/mL)	244.00 (21.54)	330.70 (78.13)	0.02

No significant differences were observed between offspring of diabetic mothers and those of control mothers in any of the conventional lipid profile parameters, including total cholesterol (TC), triglycerides (TG), HDL-C, LDL-C, and non-HDL-C (all P > 0.05 except for TG which had P = 0.05) (Table 2). Similarly, atherogenic indices such as TC/HDL-C, TG/HDL-C, TG/LDL-C, LDL-C/HDL-C, non-HDL-C/HDL-C, and the atherogenic index of plasma (AIP) were comparable between the two offspring groups (all P > 0.05) (Table 2). Furthermore, glucose homeostasis indices—namely glucose levels, the G/I ratio, insulin, and the triglyceride-glucose (TyG) index—did not differ significantly between offspring of diabetic mothers and those of control mothers (all P > 0.05) (Table 2).

**Table 2:** Comparison of biochemical profile in offspring of diabetic and non-diabetic mothers.

Parameter	Offspring of Control Mothers (n = 6)	Offspring of Diabetic Mothers (n = 6)	P value
Glucose (mg/dL)	92.83 (2.22)	95.00 (1.41)	0.07
TG (mg/dL)	61.83 (1.60)	59.33 (2.25)	0.05
TC (mg/dL)	67.17 (3.06)	66.17 (5.26)	0.69
HDL-C (mg/dL)	38.50 (2.88)	39.33 (2.25)	0.58
LDL-C (mg/dL)	16.30 (5.41)	14.97 (5.61)	0.68
Non-HDL-C (mg/dL)	28.67 (5.27)	26.83 (5.60)	0.57
TC/HDL-C	1.75 (0.19)	1.68 (0.16)	0.51
TG/HDL-C	1.61 (0.09)	1.51 (0.13)	0.17
TG/LDL-C	4.17 (1.46)	4.48 (1.73)	0.74
LDL-C/HDL-C	1.71 (0.35)	1.58 (0.24)	0.48
Non-HDL-C/HDL-C	0.75 (0.19)	0.68 (0.16)	0.51
AIP	0.20 (0.02)	0.18 (0.04)	0.21
Insulin (μIU/mL)	2.10 (0.07)	2.10 (0.03)	0.95
G/I ratio	44.19 (2.11)	45.21 (0.79)	0.29
TyG index	7.96 (0.03)	7.94 (0.03)	0.36
Asprosin (pg/mL)	234.50 (10.60)	235.00 (7.23)	0.92

### 3.2. Comparison of asprosin

**Fig 1:**
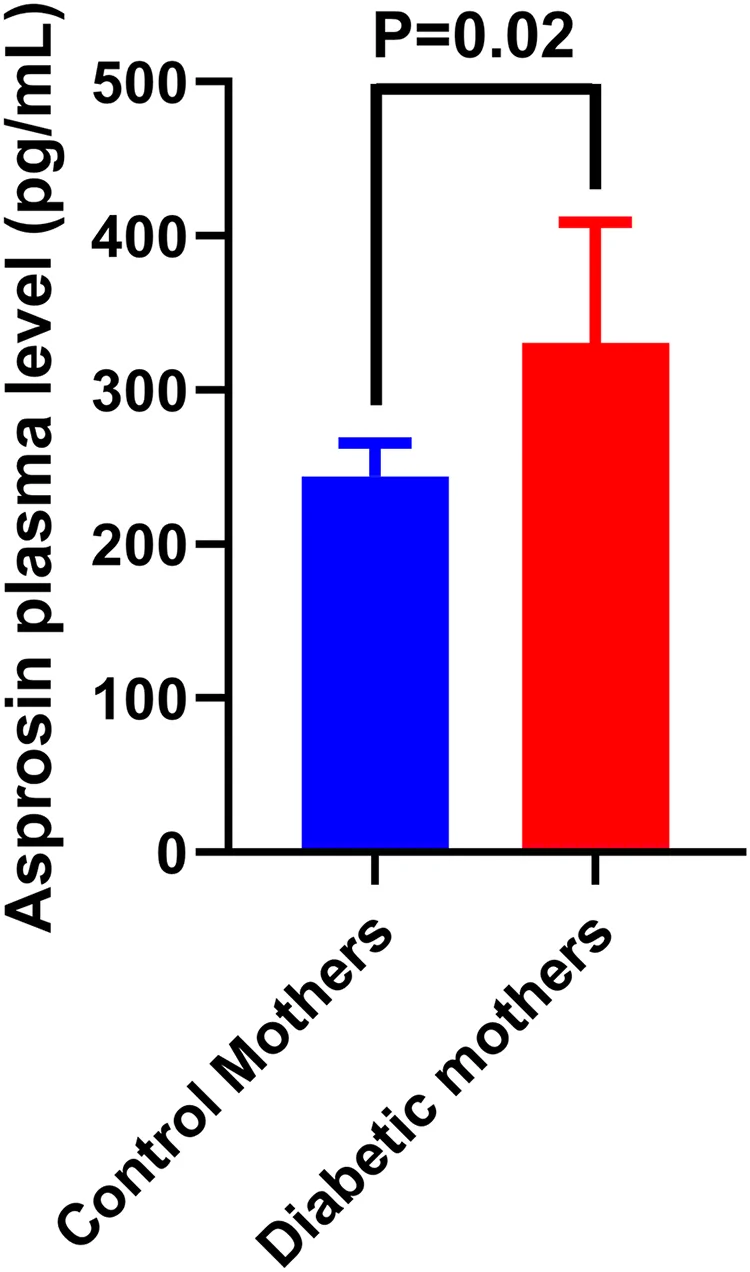
Comparison of circulating asprosin levels (pg/mL) in control group vs diabetic mothers.

**Fig 2:**
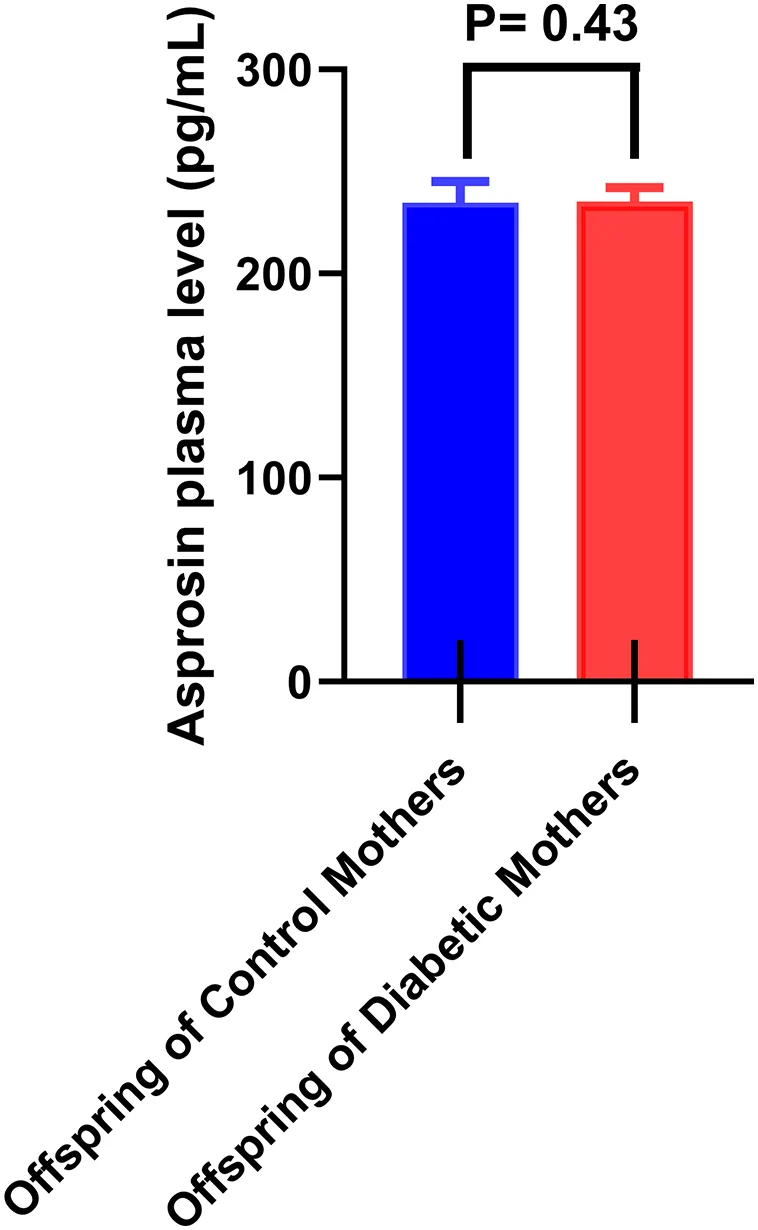
Comparison of circulating asprosin levels (pg/mL) in offspring of control mothers vs offspring of diabetic mothers.

Mothers with diabetes exhibited significantly higher asprosin levels compared to control mothers (330.7 ± 78.13 vs. 244.00 ± 21.54 pg/mL; *P* = 0.02). However, there was no statistically significant difference in asprosin levels between the offspring of diabetic mothers and the offspring of control mothers (235.00 ± 7.23 vs. 234.5 ± 10.60 pg/mL, *P* = 0.92) (Figs 1 and 2).

### 3.3. Correlation with asprosin

Due to the exploratory nature of this study and the limited sample size (n = 6 per group), all correlation analyses should be interpreted with caution. Tables 3 and 4 present the Pearson correlation coefficients between asprosin levels and various metabolic parameters in mothers and offspring. In control mothers, asprosin levels showed positive correlations with TG (r = 0.92, *P* < 0.01) and TyG index (r = 0.91, P = 0.01). In the diabetic mothers, asprosin levels showed positive correlations with glucose (r = 0.94, *P* < 0.01), G/I ratio (r = 0.93, *P* < 0.01), TyG index (r = 0.98, *P* = 0.01), TG (r = 0.96, *P* < 0.01), and several atherogenic indices, including TC/HDL-C (r = 0.90, *P* = 0.01), TG/HDL-C (r = 0.96, *P* < 0.01), non-HDL-C/HDL-C (r = 0.90, *P* = 0.01), and AIP (r = 0.97, *P* < 0.01); and significant negative correlations with insulin (r = −0.92, *P* < 0.01) and HDL-C (r = −0.96, *P* < 0.01). However, no significant correlations were found between asprosin and LDL-C, non-HDL-C, LDL-C/HDL-C or the TG/LDL-C ratio in this group.

**Table 3:** Pearson’s correlation analysis of asprosin with glucose-lipid homeostasis biomarkers in diabetic and control mothers.

Parameter	Control mothers		Mothers with DM	
	r	P value	r	P value
Glucose (mg/dL)	−0.15	0.77	0.94	<0.01
TG (mg/dL)	0.92	<0.01	0.96	<0.01
TC (mg/dL)	0.34	0.49	−0.37	0.46
HDL-C (mg/dL)	−0.29	0.56	−0.96	<0.01
LDL-C (mg/dL)	0.42	0.39	0.56	0.24
Non-HDL-C (mg/dL)	0.49	0.31	0.64	0.16
TC/HDL-C	0.55	0.25	0.90	0.01
TG/HDL-C	0.67	0.14	0.96	<0.01
TG/LDL-C	−0.08	0.86	−0.36	0.48
LDL-C/HDL-C	0.51	0.29	−0.54	0.26
Non-HDL-C/HDL-C	0.55	0.25	0.90	0.01
AIP	0.70	0.11	0.97	<0.01
Insulin (μIU/mL)	0.20	0.70	−0.92	<0.01
G/I ratio	−0.25	0.62	0.93	<0.01
TyG index	0.91	0.01	0.98	0.01

**Table 4:** Pearson’s correlation analysis of asprosin with glucose-lipid homeostasis biomarkers in offspring of diabetic and control mothers.

	Offspring of control mothers		Offspring of Mothers with DM	
Parameter	r	P value	r	P value
Glucose (mg/dL)	0.56	0.24	0.01	0.97
TG (mg/dL)	0.25	0.62	−0.29	0.57
TC (mg/dL)	−0.47	0.33	0.18	0.72
HDL-C (mg/dL)	0.009	0.98	−0.15	0.76
LDL-C (mg/dL)	−0.29	0.57	0.25	0.61
Non-HDL-C (mg/dL)	−0.28	0.58	0.23	0.65
TC/HDL-C	−0.21	0.68	0.24	0.64
TG/HDL-C	0.10	0.84	−0.05	0.91
TG/LDL-C	0.25	0.62	−0.03	0.94
LDL-C/HDL-C	−0.24	0.63	0.39	0.44
Non-HDL-C/HDL-C	−0.21	0.68	0.24	0.64
AIP	0.02	0.96	−0.05	0.91
Insulin (μIU/mL)	−0.29	0.57	−0.17	0.74
G/I ratio	0.49	0.32	0.16	0.75
TyG index	0.57	0.23	−0.33	0.52

In the offspring of both control and diabetic mothers, asprosin levels were not significantly correlated with glucose, insulin, G/I ratio, TyG index, or any of the lipid parameters measured (TG, TC, HDL-C, LDL-C, non-HDL-C, and the aforementioned lipid ratios). Given the small sample size and the absence of correction for multiple testing, these findings are considered hypothesis-generating and require validation in larger independent studies.

## 4. Discussion

### 4.1. Main findings

To our knowledge, this study is the first to investigate the impact of *in utero* exposure to maternal diabetes on offspring asprosin levels and the relationship between asprosin and glucose intolerance and dyslipidemia in these offspring. We found that mothers with diabetes had significantly higher asprosin levels compared to control mothers. Furthermore, in the diabetic mothers, asprosin levels were positively correlated with glucose, G/I ratio, TyG index, TG, and various atherogenic indices (TC/HDL-C, TG/HDL-C, and non-HDL-C/HDL-C), as well as the AIP. A significant negative correlation was observed between asprosin and HDL-C in the diabetic mothers. In control mothers, significant correlations were found between asprosin and TG and TyG. Offspring asprosin levels did not differ significantly between the two offspring groups. Additionally, there was no significant correlation between asprosin and any of the following parameters in the offspring of both groups (diabetic and control mothers): glucose, TG, TC, HDL-C, LDL-C, non-HDL-C, TC/HDL-C ratio, TG/HDL-C ratio, TG/LDL-C ratio, LDL-C/HDL-C ratio, non-HDL-C/HDL-C ratio, AIP, insulin, G/I ratio, and TyG index.

### 4.2. Comparison with previous studies

Since this is the first study to examine the effect of *in utero* exposure to diabetes on asprosin levels afterbirth, it is not possible to directly compare our findings with previous studies. However, our results align with studies that have investigated the relationship between asprosin levels and glucose homeostasis.

In agreement with our study findings, several studies have reported that asprosin levels are significantly higher in individuals with DM compared to healthy people. For example, Naiemian *et al.* (2020), found elevated asprosin levels in patients with new-onset DM [25]. Similarly, Zhang *et al.* (2020), reported higher circulating asprosin levels in people with newly diagnosed DM compared to control group [28]. Boz *et al.* (2023) reported that pregnant women with gestational diabetes had significantly higher asprosin levels than the control group [29]. Additionally, Zhang *et al*. (2020) showed that pregnant women with gestational diabetes had higher asprosin levels compared to the control group [30]. Baykus *et al*. (2019) reported a significant positive correlation between maternal and offspring asprosin levels, with higher asprosin levels observed in the umbilical cord blood of children born to mothers with gestational diabetes [15].

The relationship between asprosin and glucose and lipid profiles remain unclear. In consistence with some previous studies, we found a positive correlation between asprosin and glucose, TG, TC/HDL-C, TG/HDL-C, and AIP levels in diabetic mothers [25,28,31]. However, we did not find a significant relationship between asprosin with glucose or lipid profiles in offspring born to both control mothers and mothers with DM. These results are consistent with studies that has also failed to find significant relationships between asprosin and various metabolic parameters, including body mass index (BMI) [32,33], glucose [32,34], HbA1c(32, 34), TG [32,33,35], TC [32,33,35], HDL-C [32,33,35], LDL-C [32,33,35], insulin [32], HOMA-IR [32], in healthy individuals. However, in light of the significant correlations observed between asprosin and both TG and the TyG index, sex and/or age may represent modulatory factors that influence the metabolic effects of asprosin on glucose and lipid homeostasis. Of note, several human studies have reported sex-related differences in circulating asprosin levels between females and males.

### 4.3. Potential mechanisms

Although the exact mechanism underlying increased asprosin level in DM remains unknown, it is possible that hyperglycemia may play a role in regulating asprosin levels. Anil B. Gaikwad investigated the impact of hyperglycemic condition on the expression of the fibrillin-1 (FBN1) gene, which codes for asprosin. In a study using male Sprague-Dawley rats, they induced DM and insulin resistance by feeding a high-fat diet (HFD) and administering a low dose of streptozotocin (STZ). The results showed that hyperglycemia triggers dephosphorylation and deacetylation of H3, leading to epigenetic changes that alter the expression of the FBN1 gene [27].

### 4.4. Limitations

While this study represents the first investigation into the impact of *in utero* exposure to diabetes on asprosin levels in offspring and the role of asprosin in glucose and lipid dysregulation in offspring exposed prenatally to diabetes, several limitations should be considered when interpreting the findings. Firstly, the use of rats as an animal model may limit the generalization of the obtained results to humans [36]. Secondly, the type of diabetes may influence the results [37]. Thirdly, our findings were obtained exclusively in Wistar rats. Given that genetic background can influence metabolic programming and adipokine responses, the generalizability of our conclusions to other rat strains or to humans remains unknown. Future studies using multiple strains are warranted to determine whether the lack of change in offspring asprosin is a strain‑specific phenomenon in Wistar rats or a relatively universal feature of intrauterine exposure to hyperglycemia. Fourthly, only male offspring were studied; therefore, the findings may not be generalizable to females, and sex-specific effects of in utero hyperglycemia on asprosin regulation cannot be ruled out. Lastly, the relatively small sample size in this study warrants further investigation with a larger number of samples [38]. Therefore, the lack of a significant difference between offspring of diabetic mothers and healthy controls cannot be interpreted as definitive evidence that maternal diabetes has no effect on offspring asprosin. The possibility of a small effect that could not be detected with the current sample size remains. Future studies with larger sample sizes are necessary to clarify the potential role of asprosin in this context.

## 5. Conclusion

Our findings demonstrate a significant elevation of asprosin levels in mothers with diabetes compared to control mothers. However, *in utero* exposure to diabetes did not result in significant different offspring asprosin levels. Furthermore, we found no significant correlations between asprosin levels and glucose or lipid levels in either the diabetic or control offspring groups. Interestingly, positive correlations between asprosin and glucose/lipid parameters were observed in the diabetic mothers, but not in the offspring of control and diabetic mothers. These results suggest that while maternal asprosin levels are affected by diabetes, *in utero* exposure to diabetes does not appear to have a lasting effect on offspring asprosin levels, and that asprosin may not be a key mediator in the development of glucose intolerance or dyslipidemia in the offspring of diabetic mothers.

### Key findings

- Asprosin levels were significantly higher in mothers with diabetes compared to the control group.
- No significant difference was observed in asprosin levels between offspring born to mothers with diabetes and those in the control group.
- In mothers with diabetes, asprosin showed a significant positive correlation with TG, and various atherogenic indices including TC/HDL-C, TG/HDL-C, and non-HDL-C/HDL-C, as well as the AIP. Additionally, a significant negative correlation was found with HDL-C.
- In mothers with diabetes, asprosin showed a significant correlation with glucose, insulin, G/I ratio and TyG index.
- In control mothers, significant correlations were observed with TG and TyG index.
- In offspring from both groups, no significant correlation was observed between asprosin and glucose homeostasis or lipid indices.

## Supporting information

S1 Fig Graphical abstract. The graphical abstract provides a visual summary of the experimental design and the main findings of the study. Briefly, pregnant rats were assigned to healthy or diabetic groups, and male offspring were followed until 16 weeks of age. Plasma samples were then collected to evaluate asprosin levels. The graphical abstract illustrates the overall study workflow and highlights the observed differences in plasma asprosin concentrations between the experimental groups.
(TIF)
